# Affective disorders in the elderly in different European countries: Results from the MentDis_ICF65+ study

**DOI:** 10.1371/journal.pone.0224871

**Published:** 2019-11-11

**Authors:** Sylke Andreas, Maria Dehoust, Jana Volkert, Holger Schulz, Susanne Sehner, Anna Suling, Karl Wegscheider, Berta Ausín, Alessandra Canuto, Mike J. Crawford, Chiara Da Ronch, Luigi Grassi, Yael Hershkovitz, Manuel Muñoz, Alan Quirk, Ora Rotenstein, Ana Belén Santos-Olmo, Arieh Y. Shalev, Kerstin Weber, Hans-Ulrich Wittchen, Martin Härter

**Affiliations:** 1 Department of Medical Psychology, University Medical Center Hamburg-Eppendorf, Hamburg, Germany; 2 Institute for Psychology, Alpen-Adria-Universität Klagenfurt, Klagenfurt, Austria; 3 Department of Psychology, University Witten, Herdecke, Germany; 4 Department of Psychosocial Prevention, University of Heidelberg, Heidelberg, Germany; 5 Institute of Medical Biometry and Epidemiology, University Medical Centre Hamburg- Eppendorf, Hamburg, Germany; 6 School of Psychology, Complutense University of Madrid, Campus de Somosaguas s/n, Madrid, Spain; 7 Nant Foundation, East Vaud Psychiatric Institute, Route de Nant, Corsier-sur-Vevey, Switzerland; 8 College Centre for Quality Improvement, Royal College of Psychiatrists, London, United Kingdom; 9 Institute of Psychiatry, Dpt. Biomedical and Specialty Surgical Sciences, Corso, Italy; 10 Department of Psychiatry, Hadassah University Medical Center, Kiryat Hadassah, Jerusalem, Israel; 11 Department of Psychiatry, NY Langone Medical Center, New York, NY, United States of America; 12 Curabilis, Medical Direction, University Hospitals of Geneva, Chemin de Champ-Dollon, Puplinge, Switzerland; 13 Institute of Clinical Psychology and Psychotherapy, Technische Universtiät Dresden, Chemnitzer Straße, Dresden, Germany; Department of Psychiatry and Neuropsychology, Maastricht University Medical Center, NETHERLANDS

## Abstract

**Objectives:**

Affective disorders are among the most prevalent disorders in the elderly. The present study aims to examine the sociodemographic and clinical correlates of major depressive disorder (MDD) and dysthymia in different European and Associated countries using standardized interview techniques. Furthermore, service utilization for the elderly with depression is assessed.

**Methods:**

The MentDis_ICF65+ study is a cross-sectional survey (N = 3,142) that was conducted in six different European and Associated countries (Germany, Italy, Spain, Switzerland, England and Israel) with a subsample of n = 463 elderly with any depressive disorder.

**Results:**

Sociodemographic and clinical correlates, such as gender, age and symptom severity, were significantly associated with MDD and dysthymia in the elderly. Only 50% of elderly with any depressive disorder were treated with psycho- or pharmacotherapy.

**Conclusion:**

Our findings identified sociodemographic and clinical characteristics for depression risk in the elderly and highlight the need to improve service delivery to older adults who suffer from depression.

## Introduction

Affective disorders are one of the most prevalent conditions among mental disorders in individuals over 65 years. In a recent meta-analysis [1], the prevalence rate varied between 3.3% for current major depression and 16.5% for lifetime major depression. Using dimensional measures for depression, the prevalence rate was 19.5%. Volkert and colleagues [1] identified only one study that employed a standardized-structured interview, which yielded a 1.3% prevalence rate of dysthymia. The wide range of prevalence rates of depressive disorder may be related to heterogeneous study designs, the use of different methodologies, including different instruments, variations in the definitions of old age and different populations. Large cross-cultural studies of depression in the elderly are scarce [2]. Therefore, the aim of the MentDis_ICF65+ study was to determine lifetime, 12-month and current prevalence estimates for a wide range of mental disorders, including depressive disorders, for elderly in different European and European associated countries using a standardized and structured interview that was specifically adapted to the needs of the elderly [3]. In our study, the MentDis_ICF65+ study, the adjusted and weighted 12-month prevalence for any depressive disorder was 13.0%, and the prevalence rates significantly varied across centers [3]. The highest rate was identified in Switzerland at 28.7% and the lowest rate was identified in Italy at 9.1% [3].

Depressive disorders are associated with many sociodemographic and clinical factors, such as gender, marital status, comorbidity, physical illness and stressful life events [4]. Evidence indicates that elderly women are at a higher risk for depression than older men [5,4,6]. Furthermore, older individuals who suffer from depression are often chronically physically ill, express cognitive problems and are functionally impaired [4]. In a review, Djernes [4] identified additional risk factors for depression in the elderly: a lack of or loss of close social relationships, a previous history of depression, widowhood, stressful life events (e.g., loss of a significant other), housing in institutional care, low income and lack of social activities. In a more recent study of an older community-dwelling Australian population, Almeida and colleagues [7] identified various risk factors associated with depression in older adults, such as adverse childhood experiences (e.g., early sexual abuse), adverse lifestyles (e.g., smoking), health hazards (e.g., alcohol abuse), comorbidity (e.g., diabetes) and socioeconomic characteristics (e.g., financial stress). Furthermore, depression is an important risk factor for suicide mortality in the elderly [8,9].

Depression is a very serious disorder; however, under-recognition and under-treatment in the elderly are severe problems. Some authors [4,10] showed that the concordance between a clinician-assessed depression diagnosis in primary care settings and depression assessed with a structured clinical interview in older adults is only approximately 18%.

As a result of the heterogeneity of the examined samples in previous studies and the paucity of cross-cultural studies, it is difficult to generalize prevalence rates and impairments for mental disorders across countries. Thus, our study aims to investigate the sociodemographic correlates, comorbidity patterns, the relationship to symptom severity and quality of life, disability and impairment. More specifically, we examine the following research questions in a community-dwelling sample of 65-84-year-old participants from different European countries:

1. How frequently are major depression and dysthymia associated with sociodemographic correlates and other mental disorders and general medical conditions?
2. How impairing are major depression and dysthymia?
3. What services are used by elderly individuals with major depression or dysthymia?

## Methods

The findings presented in this study are part of the MentDis_ICF65+ project on mental disorders in the elderly, a representative, stepwise, cross-sectional survey conducted in different European and Associated countries (Germany, Italy, Spain, Switzerland, England and Israel). The design of this multi-center study has been described in detail elsewhere [11].

### Sample

The complete details of the sampling procedure are also provided by Andreas and colleagues [3]. A sample of n = 3142 older men and women aged 65–84 years living in selected catchment community areas of each participating country stratified by age and gender was randomly drawn from the population registries in Germany and Italy and postal addresses of market research companies in Spain, Switzerland, England and Israel. The inclusion criteria for the participants included the ability to provide written informed consent, living in the predefined catchment area at the beginning of the cross-sectional study and an age between 65 and 84 years. The exclusion criteria included severe cognitive impairment as assessed with the MMSE (Mini-Mental State Examination, Mini cut-off score > 18) [12] and an insufficient level of corresponding language. A harmonized procedure in contacting each participant and conducting the survey was realised, including initial contact by phone and mail, standardised interviewer training, implementation of a standardised study protocol for all test centres, and using stringent, high-quality data-control procedures. The response rate varied by country, age and gender. As described in previous publications [3,13], responder analyses showed significant differences in the response rate between the centres and age groups but not between genders. The age effect indicates that the response rate was significantly higher for younger participants than for older participants. The overall response rate of our study was 20%, which is comparable with that of previous studies with similar recruitment procedures [14]. Furthermore, representativeness analysis showed that the differences were small between the catchment areas in our study compared with catchment areas of the overall population of the participating countries with regard to sociodemographic characteristics (such as work status, marital status and education) [13]; however, these differences were significant because of the large size of the databases. Furthermore, the minor differences that were identified are not clinically relevant. The study was approved by research ethics committees in all six centers (Germany: Hamburg Ethics Committee of the Medical Association No. 2895; Italy: Ferrara No. 0096637 5/11/2009; Israel: Jerusalem No. 0376-09-HMO; Spain: Madrid No. 22032010; Switzerland: University Hospitals of Geneva ethics committee, Protocol No. 09–121; and UK: National Research Ethics Service No. 10/H0715/21) [11].

### Measures

#### Assessment of mental and physical disorders

Computer assisted face-to-face interviews with an adapted, age-specific version of the Composite International Diagnostic Interview (CIDI65+) [15] were conducted by trained lay interviewers with household residents between January and October 2011. The interview covers a wide range of mental health problems, such as anxiety disorders (panic, panic disorder, generalized anxiety disorder, agoraphobia, social and specific phobias), affective disorders (major depressive disorder, dysthymia and bipolar disorders), psychotic symptoms, obsessive-compulsive disorder, substance abuse (screening sections for nicotine, alcohol, and drugs/medication), and somatoform disorders, as well as acute stress- and posttraumatic stress disorders. Moreover, cognitive impairment, somatic morbidity and the use of health care services are assessed.

Within the CIDI65+ section on depressive disorders, a general depressive syndrome is initially assessed, and the different subgroups of depressive disorders are probed by additional questions during the further course of the section. The section starts with a comprehensive list of 31 symptoms that cover all DSM-IV [16] criteria for depression (Table 1). In a first step, all current symptoms coded as having been experienced “almost always” or “at least 50% of the time” in the past 4 weeks are considered, followed by a re-evaluation of the symptom list with regard to lifetime morbidity (Table 1). Symptoms are subsequently explored in depth, including the assessment of time criteria, as well as clinically significant impairments in social, everyday work, or other important areas of functioning. The CIDI65+ incorporates diagnostic algorithms for major depressive disorder and dysthymia, in which organic exclusions and diagnostic hierarchy rules are applied in the diagnostic process [15]. Wittchen et al. [15] tested the reliability of the age-sensitive CIDI65+ and stated that the instrument is reliable for assessing most mental disorders, distress, impairment and time-related information in the elderly.

**Table 1:** List of depressive symptoms assessed within the Composite International Diagnostic Interview (CIDI65+).

Life-time	During the past 4 weeks, how often were you…	almost always	about 50% of time	occa-sionally	almost never
	*A Sadness/depressed*				
	feeling sad, miserable or depressed	○	○	○	○
	*B Loss of interest and energy*				
	having no interest in things that you usually enjoy	○	○	○	○
	*C Nervous*, *worried*				
	feeling irritable, annoyed or in bad mood	**○**	○	○	○
	*D Appetite and weight*				
	experiencing a loss of appetite or weight	○	○	○	○
	*E Sleep*				
	having trouble falling asleep	○	○	○	○
	*F Slowness/restlessness*				
	talking or moving more than is normal for you	○	○	○	○
	*G Feelings of guilt/worthlessness*				
	blaming yourself or feeling guilty	○	○	○	○
	*H Concentration*				
	having trouble concentrating on important things	○	○	○	○
	*Suicidality*				
	Did you ever actually attempt to end your life or commit suicide?	○	○	○	○

#### Assessment of quality of life, level of functioning and symptom severity

In addition to the CIDI65+, two self-rated questionnaires used to assess quality of life and functioning were deployed. The WHO Quality of Life BREF [WHOQoL-BREF] [17] is a widely used instrument with good psychometric properties [18] and can be successfully administered in older individuals [19]. The widely used 12-item self-administered version of the WHO Disability Assessment Schedule II [WHODAS-II] [20] was used to assess the level of functioning with regard to cognition, mobility, self-care, getting along, life activities and participation. As part of the WHODAS, the number of days in the past month a respondent was completely unable to carry out his or her usual activities or work because of a health condition (i.e., disability days) was also assessed. To rate the symptom severity, the Health of the Nation Outcome Scales 65+ [HoNOS65+] [21] was administered. This expert-rated instrument, which was used in our study by interviewers who had received HoNOS training, consists of 12 scales that measure severity with regards to behavior, impairment, symptoms and social functioning. These scales include: behavioral disturbance, non-accidental self-injury, problem drinking or drug use, cognitive impairment, physical illness, hallucinations and delusions, depressive symptoms, other mental and behavioral symptoms (including somatoform symptoms), problems with relationships, problems with activities of daily living, problems with living conditions and problems with leisure activities. Each item is scored from 0 (no problem) to 4 (severe problem) on a 5-point scale. A review of the psychometric properties of the HoNOS65+ concludes that the instrument has good validity, reliability, sensitivity to change, and utility [22].

### Statistical analyses

All analyses were computed using Stata 12.1. [23] Analyses take into account the survey structure of the collected data and are weighted in reference to the number of inhabitants of the countries included in the present study. Adjusted prevalence rates for “any depressive disorder”, including major depression and dysthymia, were estimated as marginal means from a logistic regression. Further logistic regressions were calculated to explore the associations between depressive disorders and sociodemographic factors (age, gender, marital status, financial situation, and education) and identify patterns of co-morbidity. To analyze the relation between depressive disorders and measures of functional impairment, quality of life and symptom severity, we performed separate linear regression analyses with the WHODAS II sum score, disability days, WHOQoL-BREF global score, and HoNOS65+ total score as dependent variables; these models were adjusted for gender, age, and any other mental disorder. In addition, we present descriptive analyses of mental health service use and use of medication.

## Results

### Sociodemographic and clinical correlates of depressive disorder

The socioeconomic correlates of past-year depression and past-year dysthymia are shown in Table 2. We found a significant gender difference for both major depression and dysthymia, in which men were at a lower risk of suffering from these disorders (OR_major depression_ = 0.50, 95%-CI [0.32; 0.91]; p<0.01; OR_dysthymia_ = 0.58, 95%-CI [0.36–0.94]; p<0.05). Compared with their younger counterparts (65 to 74), the participants aged 75 to 79 years (OR = 0.45, 95%-CI [0.28; 0.72]; p<0.01) and aged 80–84 years (OR = 0.49, 95%-CI [0.24; 0.98]; p<0.05) exhibited a lower likelihood of suffering from past-year major depressive disorder. This was also the case for dysthymia, in which the likelihood of suffering from this disorder was only reduced for interviewees aged 80 years or older (OR = 0.33, 95%-CI [0.12; 0.92]; p<0.05). There was no significant interaction between gender and age. When controlling for age, gender and study center, a lower level of education was identified as a significant correlate for dysthymia (OR = 0.92, 95%-CI [0.86; 0.97]; p<0.01). Moreover, the participants who rated their religious affiliation as being “somewhat important” had a lower risk of suffering from past year major depression compared with the participants who rated it as “very important” (OR = 0.68, 95%-CI [0.47; 0.98]; p<0.05). No further sociodemographic correlates were identified (Table 2).

**Table 2:** Sociodemographic correlates of past-year major depressive disorder and past-year dysthymia.

	12-month major depression (n = 372)			12-month dysthymia (n = 104)		
	n (% | %_ad_)	OR (95% CI)	p	n (% | %_ad_)	OR (95% CI)	p
**Gender**						
female	252 (15.83 | 13.06)	REF	0.758	70 (4.4 | 3.58)	REF	**-**
male	120 (7.74 | 7.09)	0.50 (0.32–0.91)	**<0.01**	34 (2.19 | 2.13)	0.58 (0.36–0.94)	**<0.05**
**Age**						
65–69	126 (13.78 | 13.48)	REF	-	32 (3.49 | 3.86)	REF	-
70–74	112 (14.05 | 12.78)	0.94 (0.57–1.53)	0.789	27 (3.39 | 2.95)	0.75 (0.38–1.49)	0.392
75–79	75 (8.94 | 6.76)	0.45 (0.28–0.72)	**<0.01**	32 (3.81 | 3.37)	0.86 (0.44–1.67)	0.645
>80	59 (10.03 | 7.24)	0.49 (0.24–0.98)	**<0.05**	13 (2.21 | 1.34)	0.33 (0.12–0.92)	**<0.05**
**Marital status**						
married	193 (10.08 | 9.33)	REF	-	51 (2.66 | 2.22)	REF	
widowed/separated/divorced	146 (15.16 | 13.08)	1.49 (0.67–3.31)	0.314	53 (4.9 | 4.44)	2.08 (0.68–6.40)	0.188
never married/other	14 (9.86 | 5.69)	0.58 (0.22–1.53)	0.254	0	-	-
**Financial situation**						
very good	40(11.24 | 10.9)	REF	-	12 (3.37 | 5.71)	REF	
good	172 (12.54 | 10.86)	1.00 (0.58–1.72)	0.985	37 (2.7 | 2.95)	0.49 (0.21–1.16)	0.100
just enough	114 (9.96 | 9.31)	0.83 (0.47–1.46)	0.502	33 (2.88 | 2.35)	0.39 (0.11–1.32)	0.121
poor	35 (15.98 | 13.72)	1.32 (0.48–3.60)	0.573	15 (6.85 | 3.62)	0.61 (0.18–2.03)	0.399
very poor	7 (18.92 | 17.5)	1.79 (0.39–8.13)	0.432	6 (16.22 | 7.44)	1.35 (0.18–10.16)	0.762
**Living situation**						
not living alone	223 (10.21 | 10.46)	REF	-	62 (2.84 | 3.46)	REF	-
living alone	146 (15.38 | 10.64)	1.02 (0.94–2.13)	0.954	42 (4.43 | 2.33)	0.66 (0.26–1.66)	0.354
**Religious affiliation**						
very important	105 (12.7 | 12.96)	REF	-	19 (2.3 | 2.7)	REF	-
somewhat important	110 (11.49 | 9.33)	0.68 (0.47–0.98)	**<0.05**	39 (4.08 | 3.57)	1.35 (0.51–3.55)	0.527
not very important	71 (10.76 | 9.41)	0.68 (0.43–1.10)	0.113	20 (3.03 | 2.15)	0.79 (0.24–2.56)	0.677
not at all important	85 (12.46 | 10.45)	0.77 (0.50–1.19)	0.228	25 (3.67 | 3.36)	1.26 (0.42–3.80)	0.662
**Social support**						
Number of significant others (M/SD)	9.0 (8.7)	0.99 (0.97–1.00)	0.066	7.4 (7.9)	0.98 (0.93–1.02)	0.307
**Education**						
years of schooling (m/SD)	10.0 (2.9)	1.01 (0.96–1.05)	0.748	8.9 (3.1)	0.92 (0.86–0.97)	**<0.01**
**Socioeconomic status**						
self-rating (1–10; m/SD)	6.0 (1.7)	0.88 (0.77–1.02)	0.083	5.4 (2.1)	0.77 (0.52–1.12)	0.158

**Note:** %_ad_ = adjusted and weighted percentages, taking into account the clustered and stratified sample structure and sociodemographic correlates; OR = Odds Ratio.

### Co-morbidity

The participants with any past-year depressive disorder were approximately nine times more likely to suffer from past-year PTSD (OR = 8.66, 95%-CI [2.32; 32.38]; p<0.005), four times more likely to have a past-year anxiety disorder (OR = 4.17, 95%-CI [2.69; 6.47]; p<0.001) and approximately three times more likely to suffer from any somatoform disorder (OR = 3.10, 95%-CI [2.34; 4.11]; p<0.001). The likelihood of reporting a physical illness was approximately doubled for the participants who suffered from any past-year depressive disorder (OR = 2.34, 95%-CI [1.43; 3.84]; p<0.005). No significant relationship between any depressive disorder and alcohol dependence or abuse was found.

When the comorbidity patterns were analyzed separately for both subgroups, similar trends were identified (Table 3). The likelihood of suffering from a past-year anxiety disorder was increased approximately four times for the participants with major depression (OR = 3.74, 95%-CI [2.60; 5.36]; p<0.001) and more than four times for the participants with dysthymia (OR = 4.17, 95%-CI [2.69; 6.47]; p<0.001). The participants with major depression were approximately two and a half times more likely to suffer from a comorbid somatoform disorder (OR = 2.43, 95%-CI [1.36; 4.34]; p<0.01), and the patients with dysthymia were approximately six times more likely to suffer from a somatoform disorder (OR = 6.41, 95%-CI [2.62; 15.68]; p<0.001). The risk of suffering from PTSD was increased approximately four times for the participants with major depression (OR = 3.74, 95%-CI [1.07; 13.0]; p<0.05) and approximately fourteen times for the participants with dysthymia (OR = 14.04, 95%-CI [4.70; 41.8]; p<0.001). The patients with major depression were twice as likely to report a past-year physical illness compared with the participants without major depression (OR = 2.26, 95%-CI [1.56; 3.28]; p<0.001). No relationship was identified between physical illness and dysthymia.

**Table 3:** Association of depressive disorders with other past-year DSM-IV disorders and physical illness.

	12-month major depression (n = 372)			12-month dysthymia (n = 104)		
	n (% | %_ad_)	OR (95% CI)	p	n (% | %_ad_)	OR (95% CI)	p
**Any anxiety disorder**	121 (32.53 | 36.86)	3.74 (2.60–5.36)	**< 0.001**	43 (41.35 | 46.27)	5.23 (2.66–10.28)	**< 0.001**
**Any somatoform disorder**	37 (9.95 | 8.03)	2.43 (1.36–4.34)	**< 0.01**	22 (21.15 | 18.64)	6.41 (2.62–15.68)	**< 0.001**
**PTSD**	16 (4.3 | 3.53)	3.74 (1.07–13.0)	**< 0.05**	16 (15.38 | 11.04)	14.04 (4.70–41.8)	**< 0.001**
**Alcohol dependence or abuse**	13 (3.49 | 3.83)	0.68 (0.21–2.18)	0.494	2 (1.92 | 1.36)	0.23 (0.03–1.79)	0.150
**Alcohol dependence**	4 (1.08 | 1.2)	0.96 (0.23–3.97)	0.955	2 (1.92 | 1.37)	1.11 (0.13–9.32)	0.920
**Any physical illness**	337 (90.59 | 91.72)	2.26 (1.56–3.28)	**< 0.001**	93 (89.42 | 90.77)	1.90 (0.63–5.76)	0.242

**Note:** %_ad_ = adjusted and weighted percentages, taking into account the clustered and stratified sample structure and sociodemographic correlates; OR = Odds Ratio

### Impairments

With respect to significant clinical impairment, quality of life and symptom severity, we determined that both past-year major depression and past-year dysthymia were associated with increased values of functional impairment (p<0.01) and symptom severity (p<0.001) and a significant decrease in health-related quality of life (p<0.01), in which the respective β-coefficients were higher for the sub-groups with major depression or dysthymia (Table 4). We did not identify a significant effect for the number of disability days.

**Table 4:** Association of major depression and dysthymia with measures of impairment.

	12-month major depression (n = 372)				12-month dysthymia (n = 104)			
	no M (SD)	yes M (SD)	β (95% CI)	p	no M (SD)	yes M (SD)	β (95% CI)	p
Functional impairment								
WHODAS II sum score	17.2 (6.5)	20.1 (8.1)	1.72 (0.51–2.93)	**< 0.01**	17.4 (6.6)	22.5 (8.7)	2.95 (1.00–4.91)	**< 0.01**
disability days	1.4 (5.4)	2.5 (6.1)	0.57 (-0.49–1.62)	0.274	1.4 (5.3)	4.7 (9.3)	1.97 (-0.92–4.86)	0.170
Quality of life								
Global Rating WHOQoL BREF	67.8 (18.0)	62.0 (19.1)	-3.68 (-5.79–1.58)	**<0.01**	67.7 (17.9)	49.4 (19.5)	-13.54 (-19.45 - -7.63)	**<0.001**
Symptom Severity								
HoNOS65+ total score	0.3 (0.3)	0.4 (0.4)	0.12 (0.08–0.15)	**<0.001**	0.3 (0.3)	0.7 (0.4)	0.28 (0.18–0.39)	**<0.001**

**Note:** models are adjusted for centre, age, gender, any past-year anxiety disorder, any past-year somatoform disorder, past-year post-traumatic stress disorder (PTSD), past-year substance related disorder and physical illness, as well as major depression or dysthymia; WHODAS II = WHO Disability Assessment Schedule, WHOQoL-BREFF = WHO Quality of Life BREF; HoNOS65+ = Health of the Nation Outcome Scales 65+

### Treatment

**Fig 1:**
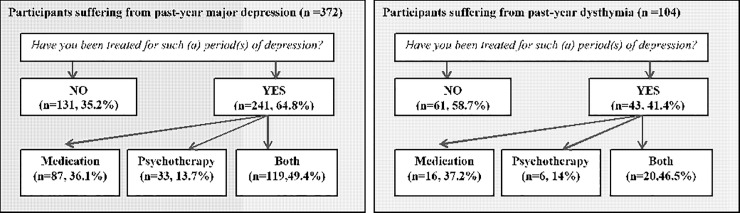
Treatments reported by participants with past-year major depression or dysthymia.

The reported service use for depression in our sample is shown in Fig 1. Overall, 64.8% of the participants with past-year major depression and 41.4% of the participants suffering from dysthymia reported having received some type of treatment. A combined treatment of medication and psychotherapy was the most frequently received treatment in both groups (49.4% in the major depression subgroup and 46.5% in the dysthymic subgroup), followed by medication alone.

## Discussion

Studies of affective disorders in the elderly that present sociodemographic correlates and associations with impairment, severity and service use in different European countries are very scarce. This investigation is the first study to use a standardized and structured clinical interview for mental disorders adapted to the needs of the elderly to report the associations of the 12-month prevalence rates with sociodemographic and clinical characteristics for affective disorders across different European and European Associated countries. Our results show that women were twice as likely as men to have had a 12-month major depressive disorder or dysthymia. The prevalence of a one-year depressive disorder decreased with increasing age. Furthermore, we identified relatively high co-morbidity rates, particularly with dysthymia and PTSD, and, as expected, associations with symptom severity and daily life activities. Surprisingly, we did not identify further correlates of major depressive disorder and dysthymia with regard to other sociodemographic characteristics, such as marital status, financial situation or living situation. We showed that elderly who rated their religious affiliation as somewhat important had a reduced risk of suffering from a major depressive disorder. Individuals who were treated for depression were most commonly treated with a combination of psychotherapy and medication.

As expected on the basis of previous results [4,24], women had twice the risk of having a depressive disorder in old age than men. Our finding that the prevalence rates of depressive disorder and dysthymia decrease with increasing age is also consistent with previous findings [25]. Surprisingly, our study indicated there were no significant differences between elderly individuals with Major Depressive Disorder (MDD) or dysthymia and those without depressive symptoms with regard to socio-demographic variables [4,26]. One potential explanation may be the choice of adjusted variables in our study compared with other studies. That is, marital status is not a relevant predictor when examined in a model adjusted for other sociodemographic factors. Another explanation may be the rather small sample size for depressive disorders. We identified an increased prevalence rate for the group of widowed, divorced, and separated elderly individuals; however, the result was not significant. Further possibilities of interpretation would refer to the process of adaption that contained a more adequate assessment of depressive symptoms in old age. Therefore, it would be possible to have included individuals with rather mild impairment in the sample of elderly individuals with MDD and dysthymia and that the relationship between marital status and depression does not emerge in this group of individuals. This would be in accordance with the empirical results of other studies in old age, in which no higher probability for depression in elderly individuals who live alone was identified. Other authors argue that the loss of a spouse is a predictable life event in old age and thus not associated with depressive symptoms [27].

Furthermore, we identified a moderate religious affiliation as an associated factor with the existence of a major depressive disorder. However, there was no significant association between religious affiliation and dysthymia. The findings regarding religious affiliation and affective disorder in the elderly and in adulthood are very heterogeneous [28–34]. Some studies found also no association between religiosity and depression [28,29]. However, when an association appears it was mostly a negative association between religiosity and depression [28,30]. Strawbridge et al. [28] reported no relationship between non-organizational religiosity (e.g., frequency of activities) and depression, but there was a negative relationship between organizational religiosity and depression like the study by Chaaya et al. [32]. Furthermore, some studies examined whether religiosity could be a protective factor for depression in the elderly [28,31]. Hsu [33] found similar results to our study. Praying activities were associated with more depressive symptoms over time. We measured religious affiliation on a 4-point Likert scale from very important to not at all important. In comparison to other studies reporting on religious activities, spirituality and well-being in the elderly, this assessment might be too unspecific. However, empirical findings are very heterogeneous [34] which indicates the need for further research on this topic.

We found a significant relationship between educational level and dysthymia, which has been previously described in a systematic review by Djernes [4,35]. In contrast to other empirical studies, [4,35] we were unable to show a significant relationship between financial status and depressive symptoms, which was likely a result of our small sample size.

As expected, we identified high rates of co-morbidity with other mental disorders (such as anxiety and somatoform disorders), as well as physical disorders [36]. Surprisingly, we determined that the risk of suffering from PTSD is 14 times higher for elderly individuals with dysthymia than for those without this disorder. This finding may be related to the composition of countries in our study. In Jerusalem (Israel), a significantly higher prevalence rate for PTSD was identified than other European countries [3]. However, our results are also consistent with a study by Glaesmer and colleagues [37]. The unweighted prevalence rates of depressive symptoms in the presence of PTSD in a German representative sample of elderly individuals were between 3% and 18% across age-groups (3% for 65- to 69-year olds and 18% for 75- to 79-year olds). Furthermore, the Depression and Early Prevention of Suicide in General Practice study (DEPS-GP) provided a risk matrix in which adverse childhood experiences (e.g., sexual or physical abuse) were associated with depression in older age [7].

In accordance with the empirical findings in the literature [38–41] we determined that the presence of depressive symptoms significantly impairs quality of life and daily routines. As expected, the expert-rated symptom severity showed significantly higher severity for elderly individuals with MDD or dysthymia than for elderly individuals without these disorders.

Overall, these findings highlight a pressing need to provide treatment for elderly individuals with depressive symptoms. Approximately 50% of individuals in both disorder groups were treated with psychotherapy or medication, whereby most individuals are treated with medication alone. As empirical results of meta-analyses have indicated that antidepressants are mostly effective to treat elderly depressed individuals [42], our results are expected. However, psychological interventions have also proven effective in treating older adults with depression. Cuijpers and colleagues [42] determined that cognitive behavior therapy and problem-solving therapy were mostly effective compared with other forms of psychotherapy (e.g., psychodynamic therapy) to treat elderly depressed individuals; however, the small number of studies makes this finding premature. The low rates of received psychological treatment in both diagnostic groups (approx. 14%) in care-as-usual indicates a need for further research and development in geronto-psychotherapeutic care.

Follow-up studies should identify factors associated with these low rates. It is possible that factors may be associated with the beliefs and attitudes of elderly individuals towards specialist provision of care. However, specialist provision of care tailor-made for the needs of elderly individuals may not exist at this point.

The study has several limitations. First, individuals with serious cognitive impairment were excluded from the study. This exclusion may have influenced the severity of the studied sample and may be the reason why some results were not significant (such as marital status and financial status). Furthermore, major differences in the prevalence rates of MDD and dysthymia existed between the countries, and different health care services for elderly individuals are offered. Therefore, the results must be interpreted under a country-specific aspect.

Despite these limitations, we assume that elderly individuals with depression are more impaired than elderly individuals without a depressive disorder. Furthermore, our results show that adequate interventions for the majority of older depressed individuals are lacking. One major strength of this study stems from the development and use of a reliable and valid age-specific structured-standardized interview, which has resulted in higher prevalence rates that illustrate the need for policy for one of the most frequent mental disorders in old age.
